# In-hospital costs of an admission for adhesive small bowel obstruction

**DOI:** 10.1186/s13017-016-0109-y

**Published:** 2016-10-06

**Authors:** Pepijn Krielen, Barend A. van den Beukel, Martijn W. J. Stommel, Harry van Goor, Chema Strik, Richard P. G. ten Broek

**Affiliations:** Department of Surgery, Radboud University Medical Center, P.O. Box 9101, 6500 HB Nijmegen, The Netherlands

**Keywords:** Colorectal surgery, SBO, Surgery, Adhesions

## Abstract

**Background:**

Previous research on the costs of treatment for ASBO is outdated and often based on reimbursements, rather than true healthcare provider costs of the admission and related interventions. An accurate estimate of the true costs of treatment is necessary to understand the healthcare burden and to model cost-efficacy of adhesion strategies. The aim of this study was to provide an accurate cost estimate of the in-hospital costs for treatment of adhesive small bowel obstruction (ASBO) using micro-costing methods.

**Methods:**

Consecutive patients admitted for ASBO to the Radboud University Medical Center from November 2013 to November 2015 were included. An episode of ASBO was defined as an admission for SBO with operative confirmation of adhesions or after radiological exclusion of other causes for SBO. For the purpose of generalization we used the costs of medication and interventions as provided by the Dutch Healthcare Authority and only if these were not available local hospital costs. We evaluated costs separately for operative and non-operative treatment for ASBO.

**Results:**

During the study period 39 admissions for ASBO were eligible for analysis. An operative treatment was required in 19 patients (48.7 %). Mean hospital stay for ASBO with operative treatment was 16.0 ± 11 days versus 4.0 ± 2.0 days for non-operative treatment (*P* = 0.003). A total of 12 patients developed complications, 2 in the non-operative group (10 %) and 10 in the operative group (52.6 %; *P* = 0.004). Overall costs for an admission for ASBO with operative treatment were €16 305 (SD €2 513), and for non-operative treatment € 2 277 (SD € 265) (*p* = <0.001). The highest expenditure with operative treatment for ASBO was made for ward stay (mean €7 856, SD €6 882), OR time (mean €2 6845, SD €1 434), ICU stay (mean €2 183, SD €4 305) and (parenteral) feeding costs (mean €1797, SD €2070). A table with correction coefficient to correct for differences in price levels for goods and services between different countries has been added.

**Conclusion:**

The in-hospital costs of an admission for ASBO are higher than previously thought. These costs can be used to guide hospital reimbursement policy and for the development of a cost-effective model for the use of adhesion barriers.

## Background

Adhesive small bowel obstruction (ASBO) is the most common pathology of the small bowel, and frequently results in surgical emergencies [1]. In a national audit in the UK small bowel obstructions accounted for 51 % of all emergency laparotomies [2]. In the United States both adhesiolysis and small bowel resection appeared in the top seven of emergency general surgeries, that count for 80 % of morbidity and death related to emergency surgery [3]. The supplementary data from this report confirmed that small bowel obstruction was the most common diagnosis in both procedures [3]. Part of the huge burden small bowel obstructions cause to patients and the healthcare system might be preventable [4, 5].

Post-operative adhesions are the cause of small bowel obstruction in 60 % of cases [6]. Application of an adhesion barrier during the index operation can reduce the risk of adhesion formation and subsequent clinical complications of adhesions [4]. In a meta-analyses of randomized controlled trials, application of a hyaluronate carboxymethylcellulose barrier reduced the risk of reoperations for ASBO after colorectal surgery with RR 0 · 49, 95 % CI 0 · 28–0 · 88 [4]. Despite the evidence for efficacy these barriers are seldom applied [7]. A reason why barriers are often not applied is that policy makers question their cost-effective and consider routine application too expensive [8, 9].

Remarkably, there is little data on the financial implications of adhesion-related complication such as ASBO that can guide policymakers in developing guidance for reimbursement, management, and prevention of this condition. The studies that modelled cost-effectiveness of adhesion barriers have used incomplete estimates or the negotiated reimbursement prices for treatment of ASBO, rather than true healthcare provider costs [7, 8, 10]. By using such incomplete estimates and reimbursement prices the conclusions about cost-effectiveness of barriers might be falsified. Moreover, concerns have been raised that reimbursement prices indeed are too low, resulting in a net loss for hospitals treating patients with ASBO [11].

In a recent study, the hospital costs of patients undergoing an emergency laparotomy in general were estimated at €15 500 per patient, which is on average €7 000 more than its reimbursement [11]. The estimate was based on operating room time, ICU and hospital stay, and did not take diagnostic or medication costs into account. Thus, it may still underestimate the actual healthcare provider costs. The costs of emergency laparotomies were also not specified for ASBO in this study [11]. More accurate and up to date data is necessary to provide a better guide to reimbursement policies, adhesion prevention, and unveil opportunities for cost reduction.

In the present study we modelled the costs of an admission for ASBO based on accurate data that in addition to the operating room times, ICU and ward stay comprised full detailed information on all relevant interventions made during the admission, including medication, parenteral feeding, imaging studies, and laboratory studies.

## Methods

All consecutive patients admitted with ASBO to the Radboudumc between November 2013 and November 2015 were eligible for inclusion. A waiver for ethical approval was obtained by local institutional review board after review of the protocol. To identify cases, the hospitals’ discharge registry was searched for patients with a reimbursement code for small bowel obstruction. The Radboudumc is a university teaching hospital in Nijmegen, the Netherlands, with complete electronic patient files. Electronic patient files of the identified records were reviewed for an admission for ASBO during the study period. ASBO was defined as an episode with operative confirmation of adhesions, or in the non-operative group as an episode of postoperative SBO in which other potential causes of bowel obstruction were excluded by appropriate means. Patients who were treated non-operatively received tube decompression and no oral feeding. Operative treatment of ASBO consisted of an explorative laparotomy with cleaving of adhesions and if necessary partial resection of small bowel. None of the patients had laparoscopic cleaving of adhesions. Complications were defined according to the criteria of the International Classification of Diseases, Tenth Revision, the National Nosocomial Infections Surveillance System, the Center for Disease Control and Prevention, or according to the decision of the senior medical staff of the department. Complications were categorized according to the Clavien-Dindo classification [12]. All complications categorized as Clavien- Dindo grade II or higher were reported.

### Costs

The total costs of the admission were divided in nine categories: operation (materials and occupancy of operating room), medication, radiology, laboratory, microbiology, ward stay, ICU days, feeding, and blood products administered during admission. All data needed for an accurate estimate of admission costs were derived from the electronic patients file. A standardized price list from the Dutch Healthcare Authority was used for the calculation of costs for occupancy of operating room, medication, radiology, laboratory, microbiology, ward stay, ICU stay, feeding and blood products [13]. No standardized price list was available for materials used during the operation. Therefore we used local prices for operation materials instead.

The price for occupancy of the operating room was based on the total anaesthesia time, and a standardized price of €16,70 per minute. Medication costs comprised the costs of all medications prescribed during the admission. The costs of medication were updated per April 2016 [14]. Costs for radiology, laboratory, microbiology were all calculated in accordance to the table provided by the Dutch healthcare authority [13]. The prices for ICU, ward stay, feeding and blood products were based on the 2015 version of the manual for costs research [15]. The costs of a day on the ICU were determined at €2015 per day. The costs of ward stay were determined at €435 per day. The prices for ward stay and ICU comprise honorarium for medical specialists, the costs for a resident managing the ward, nursing personnel, consumable goods, housing and overhead. The ICU price also counted for expenditures on respiratory support [15]. Oral feeding was also counted for in the price of ward stay. Additional expenditures for other types of feeding, such as parenteral feeding, were calculated separately and presented under the feeding category.

The average costs of operation materials were €155 if no bowel resection was performed and €436 if bowel resection was performed. The difference in price of materials was mainly attributable to the use of stapling devices.

A table of correction coefficients was added to allow for quick comparison of costs between different countries [16]. These correction coefficients are published by the European Union’s statistics department (EUROSTAT) and can be used to quickly adjust prices for the differences in price levels of goods and services between countries. Thus, these coefficient provide a rough estimate of the prices for treatment of ASBO in other countries than the Netherlands. For convenience the coefficients as published by EUROSTAT were adjusted setting the Dutch price levels as the reference standard.

### Data and statistical analysis

Baseline data consisted of patients age, sex, Charlson comorbidty index, ASA classification and the number of previous abdominal operations. Comparison was made between patients undergoing operative treatment and non- operative treatment using a Chi-square, Fisher’s exact test, independent *t*-test or Mann-Whitney *U* test where appropriate. Continuous variables are presented as means with standard deviation, or medians with interquartile range (25–75) if non-normal distribution. Dichotomous or categorical variables are presented as absolute numbers and percentages. *P* < 0.05 was considered significant. All analyses were performed using SPSS version 23°0 (Armonk, NY: IBM Corp).

## Results

**Fig. 1:**
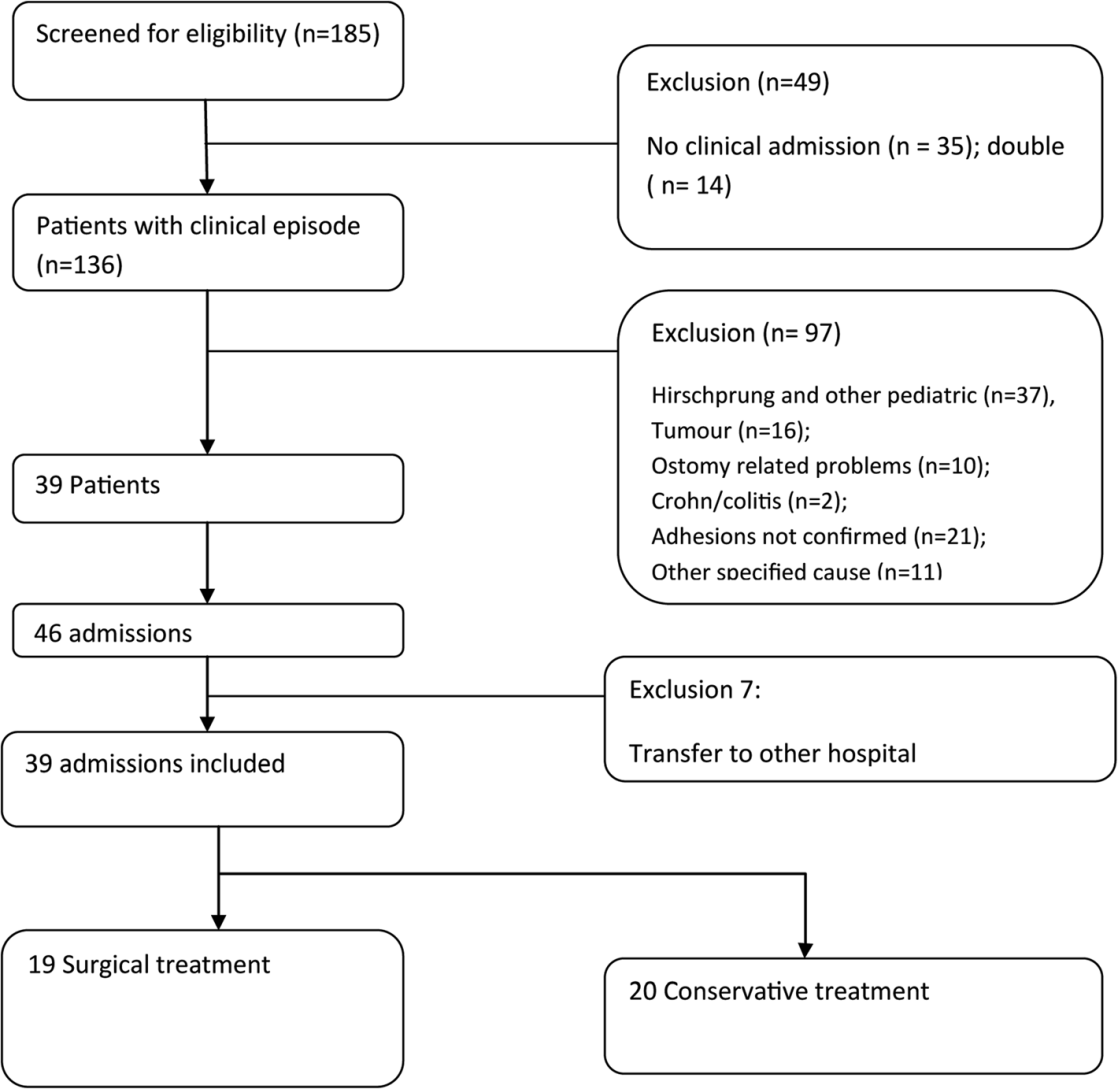
Flow chart of patients included in the study

From the hospital registry we identified 185 cases with a code of SBO. We excluded 49 patients because they were not admitted but only seen on the outpatient clinic. A total of 97 admitted patients were excluded, 29 patients were diagnosed with Hirschprung’s disease, 16 patients had a tumor, 23 had other specified cause for bowel obstruction, and in 21 patients an adhesive aetiology was unsure. Thirty-nine patients had a total of 46 admissions for ASBO during the study period. We excluded 7 admissions because patients were transferred to other hospitals for further treatment of ABSO. A total of 39 admissions of ASBO during the study period were included in the analysis (Fig. 1). Operative treatment of ASBO was required in 19 admissions (48.7 %), 20 patients were managed non- operatively (51.3 %). Indications for operative treatment was failure of non- operative management in 14 patients (73.7 %), suspected strangulation in 4 patients (21,0 %), and 1 patient had a diagnostic laparotomy (5,2 %).

Patient characteristics are shown in Table 1. There were no significant differences between groups in terms of age, sex, number of previous operations, comorbidity index, or ASA classification. Of 19 operated patients, 9 were operated within the first 24 h. Median time from admission till operation was 2 days (IQR 1–4 days). One patient was operated after 16 days. This patient had developed an adhesive small bowel obstruction after a previous appendectomy, during late pregnancy. She was treated with parenteral feeding and had explorative laparotomy delayed to be combined with caesarean section at 32 weeks of gestational age. Her bowel obstruction quickly resolved after laparotomy, and mother and child were discharged in good condition 4 days after surgery.

**Table 1:** Baseline patient characteristics

	Conservative treatment (*n* = 20)	Operative treatment (*n* = 19)	*P*-value
Age	63.6 ± 15.7	63.4 ± 14.6	0.962
Female	12 (60.0 %)	14 (73.7 %)	0.365
Previous abdominal operations^a^	2 (IQR 2-3)	1 (IQR 1-3)	0.111
Charlson Score	3.3 ± 2.4	3.6 ± 1.7	0.568
ASA score			
- Class 2	17 (85.0 %)	16 (84.2 %)	0.946
- Class 3	3 (15.0 %)	3 (15.8 %)	
Origin			
- Home	19 (95.0 %)	19 (100.0 %)	0.336
- Nursing home	1 (5.0 %)	0 (0.0 %)	

Values are presented as mean ± standard deviation or N (percentage)

^a^ median + inter quartile range

Operative treatment of ASBO led to a mean hospital stay of 16.0 days (SD 11.0 days) while non- operative treatment of ASBO led to a mean hospital stay of 4.0 days (SD 2.0 days *p* = 0.003). A total of 12 patients developed complications, two in the non- operative group (10.0 %) and 10 in the operatively treated group (52.6 %; *P* = 0.004). Complications in the non- operative group were pneumonia (*n* = 1), and *de novo* atrial fibrillation (*n* = 1). One patient in the operative group developed a staphylococcal sepsis, for which prolonged ICU admission was indicated. Other complications in the operative group comprised pneumonia (*n* = 2), wound infection (*n* = 2), intra-abdominal abscess formation (*n* = 1), *de novo* atrial fibrillation (*n* = 1), urinary tract infection (*n* = 1), bacteremia (*n* = 1), and delirium (*n* = 1). Two operative patients had a second-look laparotomy. In the first patient almost the entire small bowel was entrapped in the adhesions and appeared ischemic at the initial explorative laparotomy. Because there was doubt about the reversibility of this bowel ischemia a second look laparotomy was performed the next day, at which the bowel had normal appearance and peristalsis. The second patient underwent a second look laparotomy to inspect the anastomosis made following bowel resection at initial laparotomy. The indication for this second look was made after the patients became septic on the ICU and an anastomotic leakage was expected based on clinical evaluation. At second look on day 3 a sufficient anastomosis without signs of leakage was found. Origin of sepsis remained unsure, but a pulmonary origin was suspected after negative second look. The patient fully recovered with intravenous antibiotic treatment.

### Costs

**Fig. 2:**
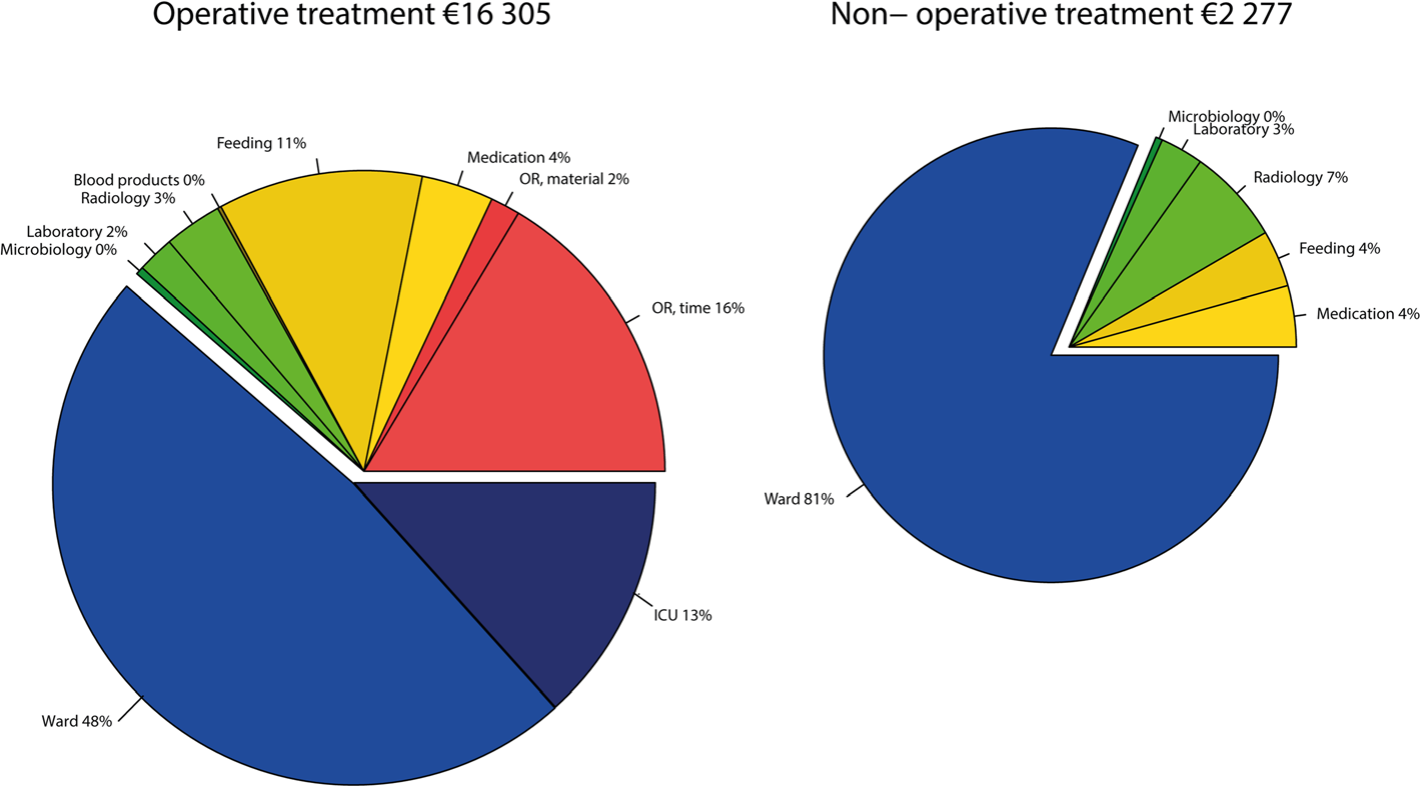
Pie charts of treatment costs for ASBO

Mean hospital stay for ASBO with operative treatment was 16.0 ± 11 days versus 4.0 ± 2.0 days for non-operative treatment (*P* = 0.003), resulting in a mean overall costs of €16 305 (SD €2 513) and 2 277 (SD €265) respectively. Mean costs were significantly different between both groups (*P* < 0.005). Costs of the different components are shown in Table 2. For both treatment strategies, ward and ICU stay was the largest component of costs (Fig. 2). The costs for operative treatment was €14 315 (SD €3 352) in uncomplicated cases and €18 095 (SD €3 776) in complicated cases, the difference was not significant. Four of the patients in the operative treatment group underwent bowel resection during laparotomy (21.5 %). Mean costs were significantly different between operative treatment with or without bowel resection, €25 395 versus €13 058 respectively. The additional operative costs for second look laparotomy in two patients were € 601 and €1 319 respectively.

**Table 2:** Comparison of costs for operative vs. non-operative treatment for ASO

	Operative		Non operative		*P*-value
	Mean	SD	Mean	SD	
Operation – anesthesia time	€ 2 684.71	€ 1 434.29	NA	NA	
Operation – materials	€ 259.90	€148.74	NA	NA	
Medication	€ 634.13	€ 816.08	€ 99.34	€ 93.17	0.011
Feeding	€ 1 797.37	€ 2 069.71	€ 91.65	€ 288.56	0.002
Blood products	€ 31.74	€ 100.79	€ 0	€ 0	
Radiology	€ 510.00	€ 467.70	€ 154.92	€ 159.88	0.003
Laboratory	€ 324.49	€ 223.20	€ 69.82	€ 38.94	0.00
Microbiology	€ 69.70	€ 98.10	€ 11.06	€ 37.34	0.023
Ward	€ 7 855.74	€ 6 881.54	€ 1 850.47	€ 913.92	0.001
ICU	€ 2 183.00	€ 4 304.93	€ 0	€ 0	
Total	€ 16 304.92	€ 2 513.07	€ 2 277.27	€ 265.34	<0.001

### Correction coefficients

An overview of correction coefficients is presented in Table 3. The correction coefficients give a global impression of differences in price levels between countries, and were standardized to the Dutch price levels. For example the correction coefficient for the United Kingdom is 1.29. This means that prices for goods and services in the United Kingdom are generally 1.29 times higher than the costs for the same goods and services in the Netherlands. The price for a non- operative treatment for ASBO in the United Kingdom are roughly estimated at 1.29*€ 2 227 = €2 872.

**Table 3:** Correction coefficients for differences in prizes of goods and surfaces

Country	Correlation coefficient
Netherlands	1.00
Australia	0.96
Austria	0.98
Belgium	0.96
Bulgaria	0.51
Canada	0.99
Croatia	0.67
Cyprus	0.80
Czech Republic	0.65
Denmark	1.24
Estonia	0.76
Finland	1.09
France	1.00
Germany	0.92
Greece	0.76
Hungary	0.60
Ireland	1.03
Israel	1.04
Italy	0.93
Latvia	0.69
Lithuania	0.64
Luxembourg	0.96
Malta	0.82
Poland	0.61
Portugal	0.77
Romania	0.56
Slovakia	0.67
Slovenia	0.75
Spain	0.86
Sweden	1.12
United Kingdom	1.29
Unites States	1.05

Source: Eurostat (http://ec.europa.eu/eurostat/web/civil-servants-remuneration/correction-coefficients) The correlation coefficients give a general estimate for the differences in price level in different countries

## Discussion

Adhesive small bowel is associated with high morbidity and costs. The average costs for a non- operative episode were over €2 000 and for a surgical episode over €16 000. The majority of costs were related to ward and ICU stay.

The costs for operative treatment of ASBO determined in this study were comparable to the €15 500 Shapter et al. reported in their estimation of the costs for an unspecified emergency laparotomy [11]. In their study, the costs for an emergency laparotomy were estimated from only the ICU stay, hospital stay, and duration of the operation. In our study these three parameters made up for only 77 % of total hospital costs in operative cases, indicating that Shapter’s estimate is too low. Local differences in price levels between the United Kingdom and the Netherlands might account for the discrepancy, implicating that true costs in the United Kingdom are higher. Indeed the correction coefficient for the United Kingdom was 1.29, indicating that goods and services are generally more expensive in the United Kingdom as compared to the Netherlands. The most important additional expenditure in operative patients is the costs for parenteral feeding. Parenteral feeding made up for 11 % of total healthcare costs in our group. The costs for operative treatment of ASBO are much higher than reimbursements for emergency laparotomies found by Shapter et al., implicating that hospitals in the United Kingdom bear a financial loss for treating patients with ASBO [11].

Correction coefficients can be used to calculate a quick estimate of the costs in a different country. However, a more precise estimate would require to recalculate the prices from the different components as listed in Table 2. An important limitation to the correction coefficients is that they are not specific for healthcare services [16]. Attempts to create more specific coefficients for healthcare have been complicated by the fact that for most condition not only the price levels of goods and services vary between countries, but also the treatment protocols itself [17]. However, we believe that it is reasonable to suggest that differences in ASBO treatment decreasing over recent years by the use of international guidelines. Adherence to the international Bologna guidelines for treatment of ASBO in this study was high [1]. As a rule, a non- operative treatment was initially instigated, unless there were signs of strangulation or ischemia. Most operative patients underwent surgery within 3 days as suggested by these guidelines.

The cost estimates in our study are useful to guide reimbursement policies and model cost-effectiveness of adhesion barriers, Because the study had high adherence to the international guidelines and the morbidity found was comparable to that reported in literature [6, 18, 19, 11]. However, the study had low power to assess the impact of factors such as complications on costs, because of the relative low sample size and retrospective nature of the study.

The relative small sample size is explained by the methodology used in this study. We included only recently admitted patients from our own institution with high ascertainment of adhesive etiology to enable the micro-costing method. Micro-costing is the gold standard for accurately defining healthcare provider costs, but seldom applied because of the large quantity of data that needs to be collected from each patient [15]. In our institution all patient data, including medication, radiology orders etc. are entered into the electronic patient file, which enabled this highly accurate method of cost estimation. For the same reasons the number of patients undergoing operative treatment was relative high in our cohort. In previous literature, non- operative treatment is successful in more than 70 % of patients with ASBO [20, 21]. We only included patients with high ascertainment of adhesive aetiology and in many of the non- operative cases the presence of adhesions could not be proved. We included only patients with high ascertainment of ASBO in this study because costs rather than treatment result was the primary endpoint. Without additional imaging or a history of previous episodes of ASBO, adhesions count for only 60 % of all cases of post-operative bowel obstruction [6]. The other 40 % might have somewhat different clinical course and costs.

We also excluded a larger number of paediatric patients with Hirschsprung’s disease. Our Institution is also a referall central for paediatric surgery. Because Hirschsprung’s disease has no separate reimbursement code in the Dutch reimbursement system, it often received the same code as used for bowel obstruction [22].

Our results show that the largest part of the expenditures in treatment of ASBO are related to the duration of hospital stay. Several studies have reported a reduced length of stay and lower incidence of postoperative ileus when adhesiolysis is performed through laparoscopy instead of laparotomy [23–25]. However, no randomized trials have been performed. In general, it will be more difficult to perform laparoscopic surgery on patients with multiple operations in history and when the bowel is very distended, increasing the risk of bowel injuries [26]. In the study of Wullstein et al. incidence of bowel injuries was higher during laparoscopic surgery for ASBO compared to open, despite a possible favourable selection in laparoscopic cases [24]. Thus, the results that laparoscopic surgery for ASBO reduces hospital stay and subsequent costs should be interpreted with caution.

The results of our study have important implications for policies regarding reimbursements. Reimbursement for operative cases of ASBO is generally too low [11]. The costs that we found for operatively treated episodes of ASBO were also much higher than the estimate Wilson applied in a cost-effectiveness model for adhesion barriers [8]. With the higher costs we found for operative cases of ASBO, it becomes more likely that adhesion barriers are cost effective in high risk procedures such as colorectal surgery. Adhesion barriers are proven to be effective in reducing the risk of reoperation for ASBO in randomized controlled trials [4, 5, 27, 28]. Moreover, a complete evaluation of cost-effectiveness of adhesion barriers should also count for other complications of adhesions, such as complications associated with adhesiolysis during repeat abdominal surgery, infertility treatments and chronic abdominal pain [8, 10].

## Conclusion

The costs of an admission for ASBO are higher than what is reported in the previous literature. Our results can be used to guide reimbursement policy and the development of a cost-effectiveness model for the use of adhesion barriers.
